# Genome-wide association study identifies candidate SNP markers and genes associated with low nitrogen tolerance in *Brassica napus*

**DOI:** 10.3389/fpls.2025.1625778

**Published:** 2025-07-07

**Authors:** Jingdong Chen, Lingli Xie, Xianfei Hou, Rui Yang, Jin Liu, Xigang Dai, Tianyuan Xue, Shuai Yin, Benbo Xu, Xuekun Zhang, Changli Zeng, Jinsong Xu

**Affiliations:** ^1^ Key Laboratory of Sustainable Crop Production in the Middle Reaches of the Yangtze River, College of Agriculture, Yangtze University, Jingzhou, China; ^2^ Hubei Engineering Research Center for Protection and Utilization of Special Biological Resources in the Hanjiang River Basin, College of Life Science, Jianghan University, Wuhan, China; ^3^ Crop Research Institute, Xinjiang Uygur Autonomous Region Academy of Agricultural Sciences, Urumqi, China

**Keywords:** *Brassica napus*, nitrogen, GWAS, stress, SNP

## Abstract

Low nitrogen (LN) stress is a major limiting factor affecting crop growth and productivity. Understanding the genetic basis of LN tolerance is essential for improving nitrogen use efficiency in *Brassica napus*. A genome-wide association study (GWAS) was conducted on a panel of 275 *B. napus* accessions using a semi-automated hydroponic system to evaluate five seedling traits–leaf number (NL), shoot length (SL), root length (RL), shoot fresh weight (SFW), and root fresh weight (RFW)—under LN conditions. The system ensured environmental uniformity and high-throughput phenotyping. Significant phenotypic variation was observed across accessions, and correlation analysis suggested that RFW and SFW are key traits associated with LN tolerance. GWAS identified 71 significant SNPs, with 20 candidate genes located near these loci. Gene Ontology analysis revealed enrichment in nitrogen compound transport functions. Several genes such as *NPF2.10*, *ATG4a*, and *AATL1* were implicated in nitrogen uptake, transport, remobilization, and stress adaptation. This study highlights the polygenic nature of LN tolerance and the importance of precise phenotyping in detecting stable genetic signals. The identified candidate genes are involved in nitrogen metabolism, autophagy, RNA processing, and amino acid transport, with transcriptomic evidence supporting the LN-responsive expression of *BnaA09G0386000ZS*. Comparative analysis with previous studies revealed unique SNP loci, likely due to differences in germplasm, nitrogen levels, and experimental design. These findings broaden our understanding of the genetic mechanisms underlying LN tolerance and provide promising targets for breeding *B. napus* varieties with improved nitrogen use efficiency.

## 1 Introduction

The balance of nitrogen (N) availability directly affects plant health and, ultimately, productivity (De Bang et al., 2020). It is estimated that nearly half of the global increase in food production can be attributed to the application of N fertilizers (Tilman et al., 2002). Approximately half of the applied N fertilizers are absorbed by crops, while the remainder accumulates in the soil (Fan et al., 2012; He et al., 2021), leading to severe ecological issues such as soil acidification and water eutrophication (Zhang et al., 2022). Therefore, identifying crop germplasm resources capable of maintaining normal growth under reduced N fertilizer application is of great significance for energy conservation, emission reduction, and environmental protection.

Rapeseed (*Brassica napus*), as an important oilseed crop, is widely cultivated in regions such as China, Northern Europe, Canada, and Australia (Raboanatahiry et al., 2021; Tang et al., 2021). Regardless of the growth type—winter, spring, or semi-winter—rapeseed exhibits a high demand for N nutrition during the seedling stage (Shen et al., 2022). However, during the maturation stage, excessive N often disrupts the balance of nutrient elements, adversely affecting plant growth and development (Ren et al., 2017). Throughout the entire growth cycle of rapeseed, N plays a pivotal role in regulating growth, significantly influencing both yield and quality (Yahbi et al., 2022).

Genome-wide association studies (GWAS), a novel genetic analysis method based on linkage disequilibrium (Alseekh et al., 2021; Tibbs-Cortes et al., 2021), have become a powerful tool for understanding the genetic architecture of complex quantitative traits in crops. With the rapid advancement of sequencing technologies, GWAS has been widely applied to decipher the genetic basis of complex traits in major cereal crops such as rice (Jiang et al., 2021; Zhong et al., 2021), wheat (Saini et al., 2021), soybean (Niu et al., 2024), and maize (Sahito et al., 2024). In recent years, the declining cost of genome sequencing has significantly reduced the cost of SNP marker development, facilitating the successful application of GWAS in detecting quantitative trait loci (QTLs) associated with complex traits in rapeseed. These traits include seed quality (Zhao et al., 2022), harvest index (Qin et al., 2022), yield (Lu et al., 2017), erucic acid content (Xu et al., 2024), glucosinolate content (Tang et al., 2023), and oil content (Pal et al., 2021). Additionally, GWAS has played a crucial role in identifying genes associated with abiotic stress tolerance in rapeseed, including those related to nutrient (Knoch et al., 2024), salt (Wan et al., 2017), drought (Khanzada et al., 2020), low-temperature (Horvath et al., 2020), and heavy metal stresses (Wan et al., 2020).

In various crops, numerous QTLs mapping studies associated with low N tolerance have been reported (Kwon et al., 2021; Yang et al., 2021; Rosa-Martínez et al., 2023; Singh et al., 2023). However, reports on the cloning of low N tolerance genes remain limited. This is primarily due to the large genomic intervals of QTL regions, making it challenging to predict and isolate candidate genes. With its high resolution, GWAS enable the rapid identification and isolation of candidate genes at significant loci, providing a significant advantage in the selection of low N tolerance genes (Ahmed et al., 2024). In the study of low N tolerance candidate genes in major crops, the application of GWAS has already achieved notable progress. For example, Li et al. (2022) utilized 230 rice germplasm accessions and combined GWAS with transcriptomics to identify 411 candidate genes associated with low N stress tolerance from five QTLs, along with 2,722 differentially expressed genes responsive to low N signals. Notably, 24 genes were identified by both methods. Sanchez et al. evaluated multiple agronomic traits in 181 doubled haploid (DH) maize germplasm under both high and low N conditions (Sanchez et al., 2023). By combining 62,077 SNP markers, the study identified several SNPs associated with agronomic traits under these conditions, providing valuable insights for the development of molecular markers for high and low N stress tolerance in maize and the creation of maize germplasm materials with enhanced N tolerance. In recent years, several studies have advanced our understanding of the genetic basis of N use efficiency (NUE) in *Brassica napus*. Li et al. (2020) conducted comparative genome and transcriptome analyses on two genotypes with contrasting NUE, revealing that differences in N uptake and assimilation, as well as coordinated carbon and N metabolism, contribute to NUE variation. Wei et al. (Yang et al., 2020) integrated genome-wide association studies (GWAS) with gene expression profiling, identifying candidate genes associated with NUE and highlighting the complex regulatory networks involved. These studies underscore the importance of integrating genomic and transcriptomic approaches to elucidate the mechanisms underlying NUE. However, variations in experimental conditions, such as N levels, developmental stages, and phenotypic traits assessed, suggest that further research is needed to uncover additional loci and genes contributing to NUE in *B. napus*. Studies using GWAS to identify QTLs and genes associated with low N (LN) stress tolerance in rapeseed have also been reported. Under LN and high N (HN) conditions, GWAS combined with RNA-seq technology detected 14 QTLs associated with N efficiency traits in rapeseed. A total of 245, 540, and 399 differentially expressed genes (DEGs) were identified as specific to LN stress, HN conditions, and common to both LN and HN, respectively. Integrating GWAS, weighted gene co-expression networks, and DEG analysis revealed 16 genes involved in root development under LN conditions (Ahmad et al., 2022). Zeng et al. used 304 rapeseed inbred lines to evaluate three related traits under LN stress, identifying 11 significant SNPs associated with LN tolerance in rapeseed. These findings provide valuable information for understanding the genetic control of LN tolerance during the seedling stage in rapeseed (Zeng et al., 2021).

To better understand the genetic control of LN tolerance traits in rapeseed, we performed a GWAS on 275 rapeseed accessions using 1,135,744 quality-controlled SNP markers derived from a high-density physical map. Using the linear mixed effects model (LMM), we identified 71 SNPs associated with five agronomic traits related to LN tolerance during the seedling stage. 20 candidate genes related to N metabolism within a 50 kb upstream and downstream region of the significant SNPs. This study provides a deeper understanding of the impact of LN stress on rapeseed seedling growth and offers valuable insights for developing molecular markers related to LN tolerance and screening LN-tolerant rapeseed materials.

## 2 Materials and methods

### 2.1 Experimental materials

The experimental materials consisted of 275 core rapeseed germplasms provided by Professor Liang Guo from the National Rapeseed Engineering Research Center, Huazhong Agricultural University. These germplasms included landraces, breeding materials, and cultivars from major rapeseed production regions in China and other parts of the world. The accession names, geographic origins, and sub-population classifications were provided in **Supplementary Table S1**. The population structure and accession information were obtained from the BnIR database (Yang et al., 2023), available at https://yanglab.hzau.edu.cn/BnIR/population_structure.

### 2.2 Hydroponic treatment

On September 8, 2021, 276 rapeseed accessions (**Supplementary Table S1**) were sown in the laboratory of the School of Life Sciences, Jianghan University, Wuhan, China. Ten trays (4×7 wells per tray) were used, and each tray and gauze were rinsed with tap water for 20 minutes, followed by a 20-minute bleach rinse. 25 seeds were sown in each well, and the trays were placed in a greenhouse for germination.

On September 15, 2021, based on plant growth status, five healthy seedlings with similar size and height were selected from each well and transplanted into a semi-automated hydroponic system constructed at the Engineering Center for Biological Resource Protection, Development, and Utilization of the Hanjiang River Basin at Jianghan University. The tanks were filled with 600 L of modified Hoagland nutrient solution (**Supplementary Table S2**). On September 23, 2021, the nutrient solution was replaced with 600 L of LN nutrient solution (N: 0.3 mmol·L^-^¹) (**Supplementary Table S2**).

On October 9, 2021, samples were collected separately from the shoots and roots. Measurements included the including leaf number (NL), shoot length (SL), root length (RL), shoot fresh weight (SFW), and root fresh weight (RFW). Three parallel replicates were set up. Three independent parallel experiments were conducted using identical hydroponic setups and conditions.

### 2.3 Genome-wide association analysis

#### 2.3.1 Marker quality control

The reference genome used for the analysis was hzau_ZS11.v10 (Song et al., 2020). A total of 26,482,024 SNPs were initially identified from the high-density physical map. Non-biallelic SNPs, those with a missing rate > 0.05, failing the Hardy-Weinberg equilibrium (HWE) test at a threshold of p < 1 × 10^-6^, and with a minor allele frequency (MAF) < 0.05, were excluded. Ultimately, 1,135,744 SNPs were retained for subsequent GWAS analysis using PLINK2 software (Chang et al., 2015).

#### 2.3.2 Genome-wide association analysis

GWAS was performed using the GEMMA software (v0.98.1) (Zhou and Stephens, 2012) within the R software. The analysis employed one model: the linear mixed effects model (LMM). Significant molecular markers were identified based on a threshold of −Log_10_ P > 5 and model criteria for association with mineral ion content. Visualization of the GWAS results, including Manhattan and Quantile-Quantile (QQ) plots, was performed using the CMplot package in R.

### 2.4 Candidate gene prediction

Based on the reference genome sequencing results, significant SNP loci identified in the GWAS analysis were mapped to the genome of *Brassica napus*. A 50 kb region upstream and downstream of these significant SNPs was considered as the candidate interval. By combining the annotation information from the *Brassica napus* genome and the molecular function annotations of genes in the Gene Ontology (GO) database (http://geneontology.org/docs/download-ontology), candidate genes related to LN stress tolerance in oilseed rape were selected. To further investigate the expression profiles of the 20 candidate genes identified in this study, we referred to a previously obtained transcriptome dataset generated using two Brassica napus lines, L168 and L435. Seeds were germinated in distilled water for 9 days, then transferred to modified Hoagland nutrient solution and grown under normal nitrogen conditions for 7 days. Subsequently, the seedlings were subjected to LN treatment (0.3 mmol/L) in a hydroponic system for 12 days at 25°C. Leaf samples were collected for RNA-seq analysis. Gene expression levels were calculated as fragments per kilobase of transcript per million mapped reads (FPKM). The differential expression of candidate genes under low-nitrogen stress was examined to support the findings of this study.

### 2.5 Data analysis

Phenotypic data were organized and analyzed using WPS Excel. Frequency distribution histograms were generated using Origin Lab 2024b. The multiple comparisons analysis was performed using SPSS 26 software.

## 3 Results

### 3.1 Phenotypic analysis of 275 *B. napus* accessions under LN stress

Under LN conditions (N: 0.3 mmol·L^-^¹), the 275 rapeseed accessions exhibited extensive genetic variation in their phenotypic traits (**Table 1**). The average leaf number (NL) was 2.70, with a range of 1.42 to 3.89, a standard deviation (SD) of 0.47, and a coefficient of variation (CV) of 17.43%. The average shoot length (SL) was 61.92 mm, with a range from 34.06 mm to 98.35 mm, SD of 9.84, and CV of 15.89%. Root length (RL) had an average of 136.42 mm, with a range from 77.06 mm to 210.96 mm, SD of 24.61, and CV of 18.04%. The average shoot fresh weight (SFW) was 0.98 g, with a range of 0.30 g to 1.95 g, SD of 0.32, and CV of 32.32%. The average root fresh weight (RFW) was 0.88 g, with a range from 0.33 g to 1.90 g, SD of 0.25, and CV of 27.90%.

**Table 1:** Phenotypic trait statistics for 275 *B. napus* accessions under low nitrogen stress.

Trait	Min	Max	Mean	SD	CV
NL (count/plant)	1.42	3.89	2.70	0.47	17.43%
SL (cm/plant)	34.06	98.35	61.92	9.84	15.89%
RL (cm/plant)	77.06	210.96	136.42	24.61	18.04%
SFW (g/plant)	0.30	1.95	0.98	0.32	32.32%
RFW (g/plant)	0.33	1.90	0.88	0.25	27.90%

**Figure 1:**
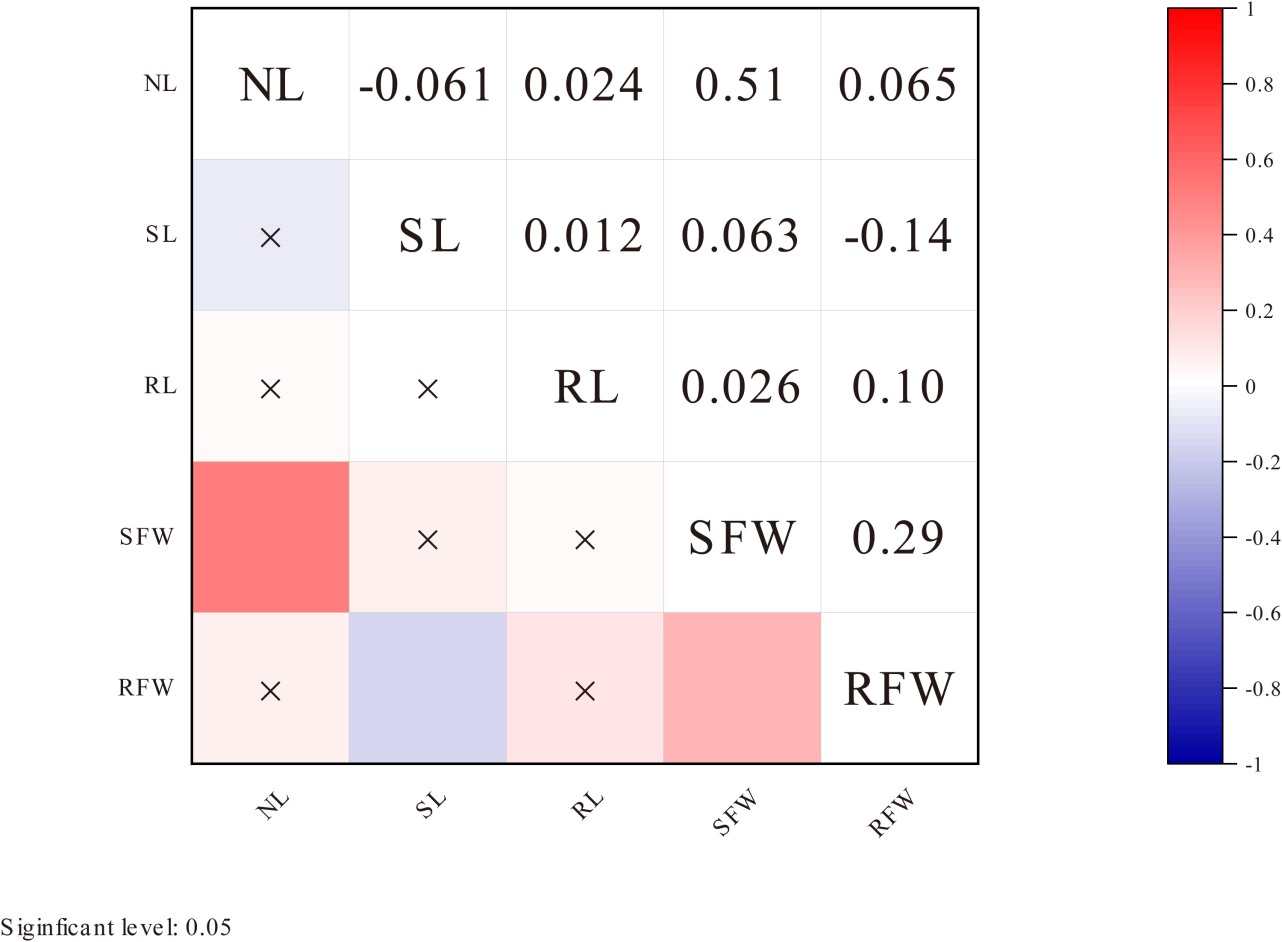
Distribution histograms of phenotypic traits for 275 *B*. *napus* accessions under low nitrogen stress.

These five traits showed continuous normal distribution (**Figure 1**) across the 275 accessions. Non-parametric tests conducted with SPSS 26 confirmed this distribution pattern. The results indicate that under LN stress, these phenotypic traits are controlled by multiple genes and are quantitative in nature.

**Figure 2:**
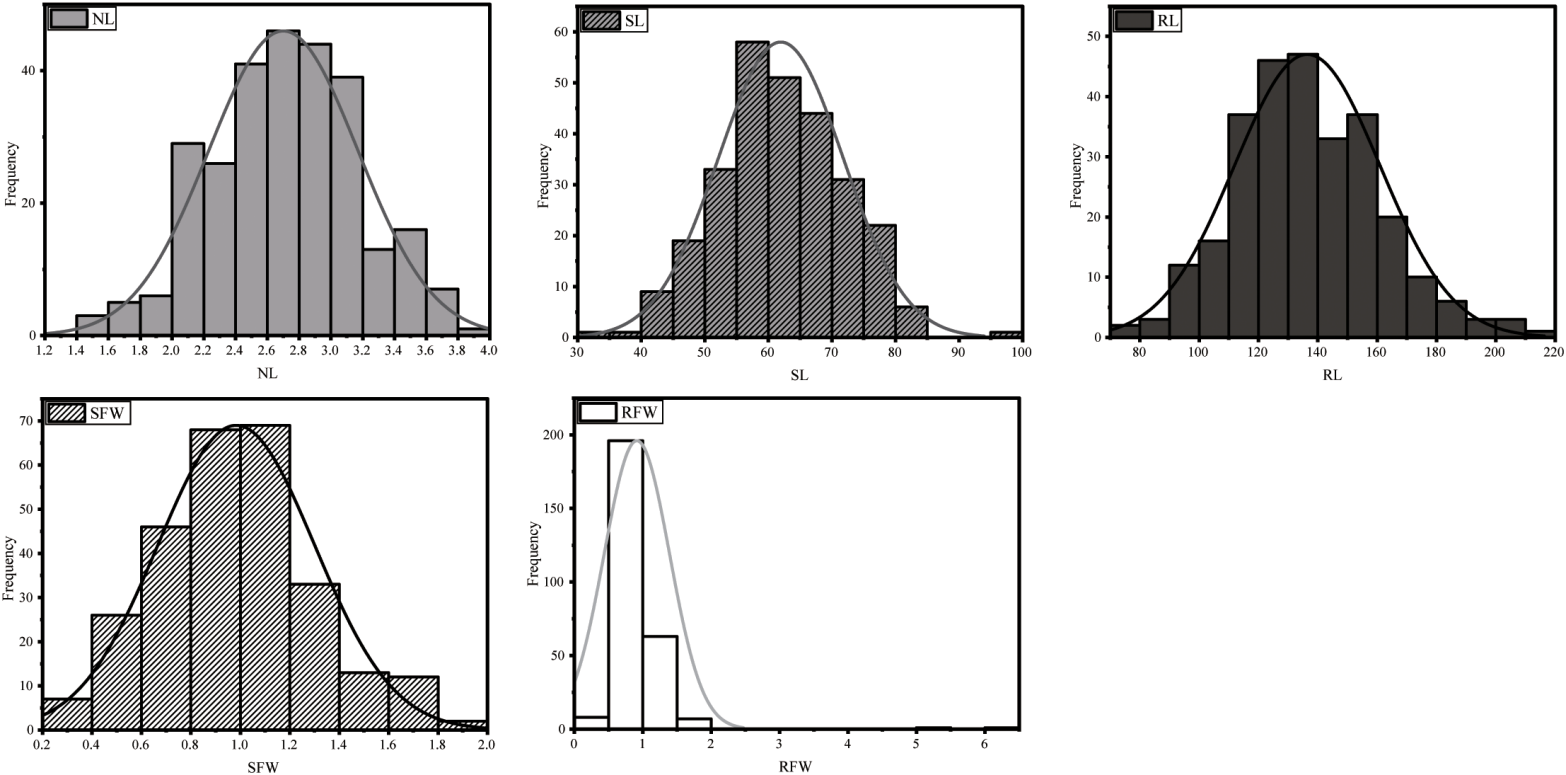
Correlation analysis of five traits under low nitrogen stress. The shading of the color blocks represents the magnitude of the correlation coefficient; the darker the color, the larger the absolute value of the correlation coefficient. The color indicates the direction of the correlation: blue represents positive correlation, and red represents negative correlation. An “×” indicates no significant correlation at the 0.05 level.

Correlation analysis results showed that at a significance level of P < 0.05, there was a significant positive correlation between NL and SFW, and between SFW and RFW under LN stress. Additionally, there was a significant positive correlation between RL and RFW, while no significant correlations were observed between other pairs of traits (**Figure 2**).

### 3.2 Significant SNPs and haplotype analysis associated with LN stress tolerance in *B. napus*

**Figure 3:**
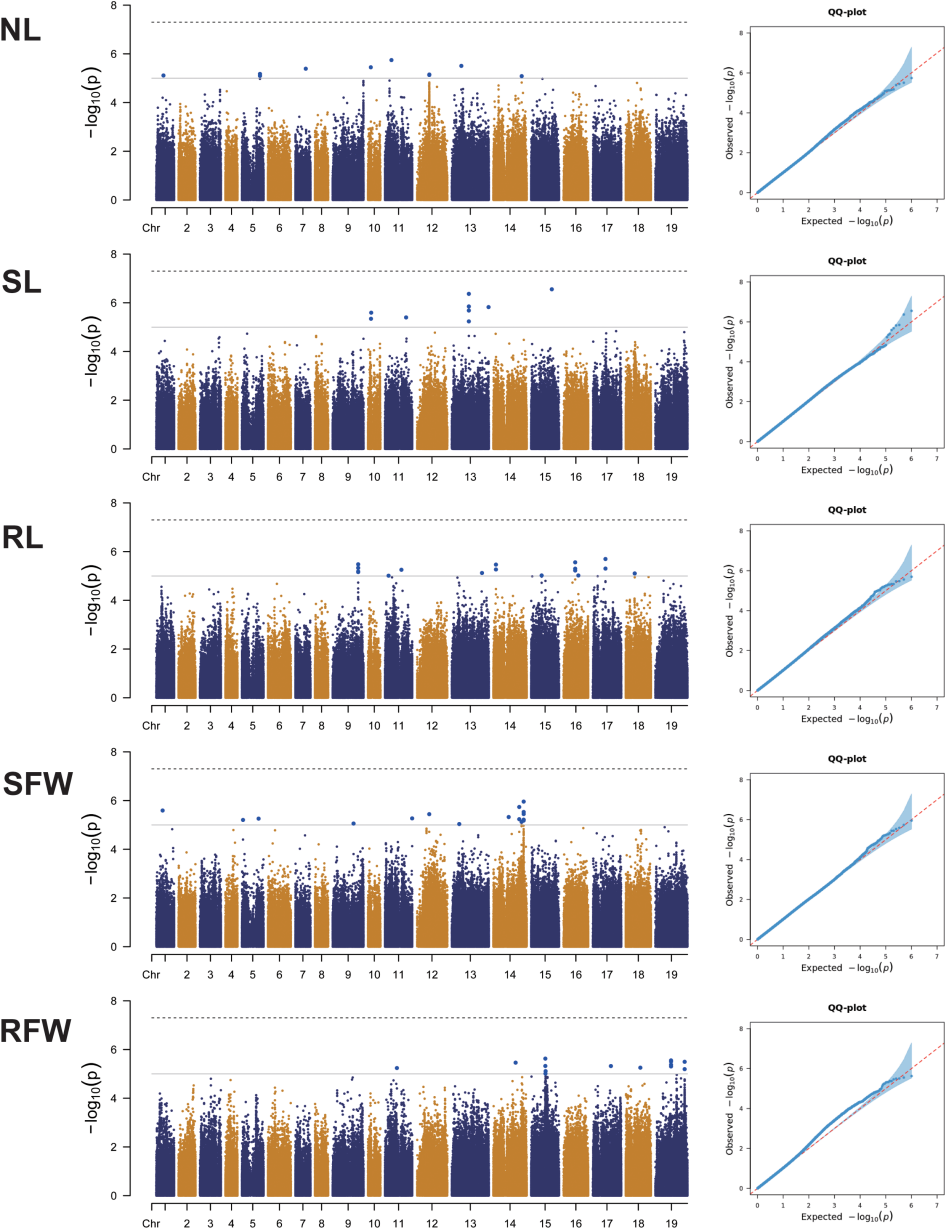
Genome-wide association analysis of five low nitrogen stress traits (NL, SL, RL, SFW, RFW) based on SNPs. Left: Manhattan plot. The dashed horizontal line represents the threshold for significant associations. The x-axis displays the physical positions of all SNPs on the *B*. *napus* chromosomes, and the y-axis shows the negative logarithmic 10-transformed p-values for each association. Right: QQ plot for the five traits.

In this study, GWAS was performed on the genotypic data of 275 rapeseed germplasm lines and five traits (NL, SL, RL, SFW, and RFW) under LN stress. As shown in the Quantile-Quantile (QQ) plot (**Figure 3**), among the linear mixed effects model (LMM), which effectively controls population structure and relatedness while handling hierarchical and complex data, reducing false positives in genetic studies.

The GWAS method revealed a total of 71 SNP markers significantly associated with the five LN stress traits (−log_10_P > 5) (**Figure 3**; **Supplementary Table S3**). These SNPs were widely distributed across all identified chromosomes of *B. napus* (**Supplementary Table S3**).

**Figure 4:**
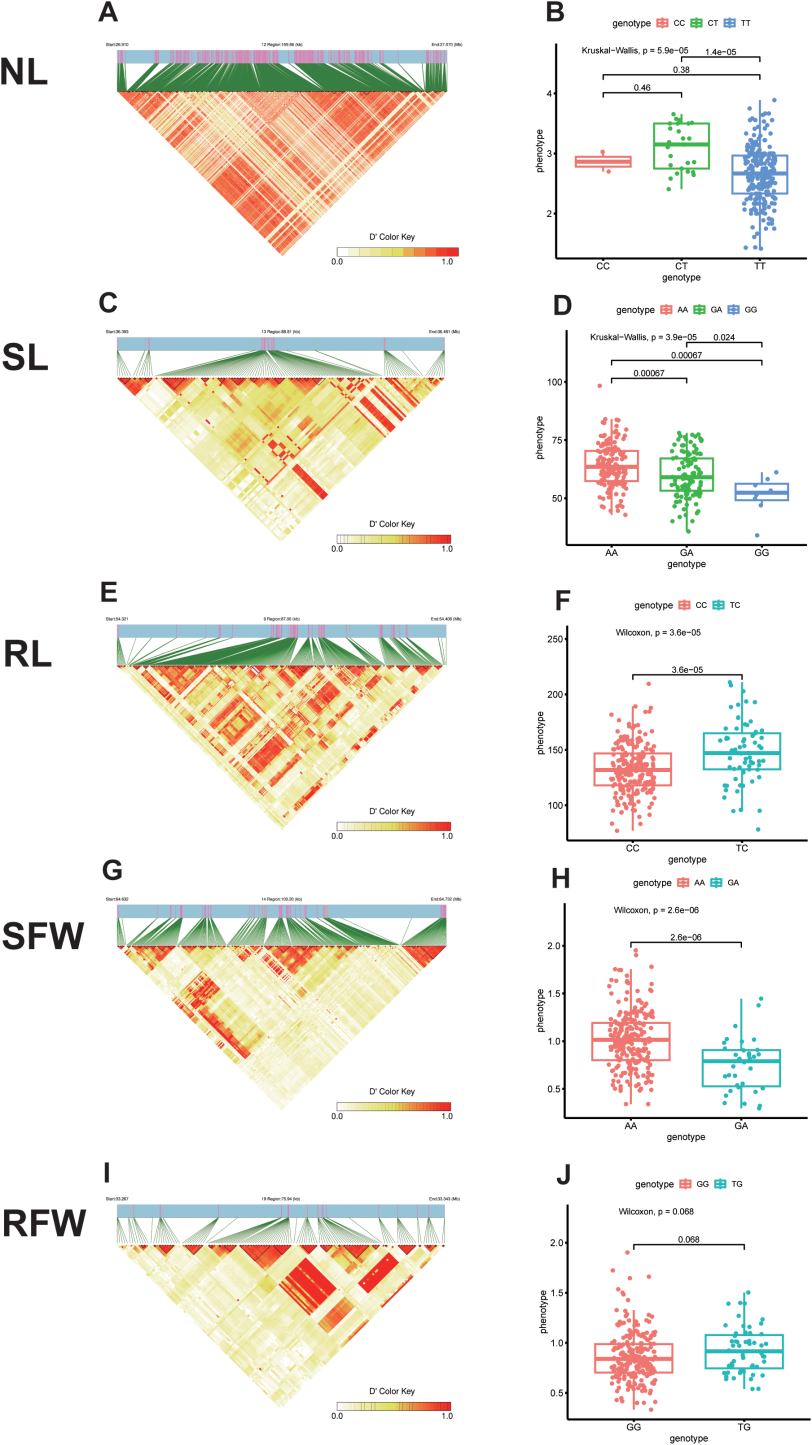
Haplotype analysis results for five traits. **(A)**Block region analysis of significant NL-related loci; **(B)** Haplotype distribution frequency analysis of significant NL-related loci; **(C)** Block region analysis of significant SL-related loci; **(D)** Haplotype distribution frequency analysis of significant SL-related loci; **(E)** Block region analysis of significant RL-related loci; **(F)** Haplotype distribution frequency analysis of significant RL-related loci; **(G)** Block region analysis of significant SFW-related loci; **(H)** Haplotype distribution frequency analysis of significant SFW-related loci; **(I)** Block region analysis of significant RFW-related loci; **(J)** Haplotype distribution frequency analysis of significant RFW-related loci.

For NL traits, three significant SNPs were identified within adjacent intervals (**Supplementary Table S3**). Haplotype analysis was performed based on the most significant SNP, scaffoldC02:27019864, revealing a haplotype block spanning 159.66 kb (C02:26959607–27019864) with three haplotypes (**Figure 4A**). After quality control of 275 rapeseed accessions, 12 accessions with poor data quality (e.g., high missing rates or significant deviations) were excluded, leaving 263 accessions for haplotype analysis. Among these, the frequencies of the three haplotypes were as follows: CC (0.76%), CT (9.51%), and TT (89.73%). CT showed significantly higher NL values than TT, while no significant differences were observed between CC-CT and CC-TT (**Figure 4B**).

For SL traits, four significant SNPs were detected within adjacent intervals. Haplotype analysis centered on the most significant SNP, scaffoldC03:36433436, identified a haplotype block spanning 88.81 kb (C03:36383435–36483479) with three haplotypes (**Figure 4C**). Following the exclusion of six accessions due to poor data quality, 269 accessions remained for analysis. The haplotype frequencies were AA (55.02%), GA (42.01%), and GG (2.97%). Significant differences in SL values were observed among the three haplotypes, with AA showing the highest values, followed by GA and GG (**Figure 4D**).

For RL traits, six significant SNPs were found within adjacent intervals. Haplotype analysis of the most significant SNP, scaffoldA09:54368129, revealed a haplotype block spanning 87.00 kb (A09:54318108–54418135) with two haplotypes (**Figure 4E**). Three accessions with poor data quality were excluded, leaving 272 accessions for analysis. The haplotype frequencies were CC (76.10%) and TC (23.90%), with TC exhibiting significantly higher RL values than CC (**Figure 4F**).

For SFW traits, five significant SNPs were identified within adjacent intervals. Haplotype analysis based on the most significant SNP, scaffoldC04:64684991, revealed a haplotype block spanning 100.20 kb (C04:64631496–64734991) with two haplotypes (**Figure 4G**). Seven accessions with poor data quality were excluded, resulting in 268 accessions for analysis. The haplotype frequencies were AA (86.94%) and GA (13.06%). AA was significantly associated with higher SFW values compared to GA (**Figure 4H**).

For RFW traits, five significant SNPs were identified within adjacent intervals. Haplotype analysis centered on the most significant SNP, scaffoldC09:33307074, identified a haplotype block spanning 75.94 kb (C09:33257073–33357104) with two haplotypes (**Figure 4I**). After excluding one low-quality accession, 274 accessions were used for analysis. The haplotype frequencies were GG (79.93%) and TG (20.07%), with no significant differences in RFW values observed between the two haplotypes (**Figure 4J**).

### 3.3 Screening of candidate genes associated with LN stress tolerance in *B. napus*

Based on the GO enrichment analysis results (**Supplementary Table S4**), we focused on genes related to plant N metabolism (GO:0071705, N compound transport) and identified 20 candidate genes associated with LN stress tolerance traits in *B. napus* (**Table 2**). Among these, one candidate gene was identified for NL (*BnaA05G0389700ZS*), one for SL (*BnaC01G0388100ZS*), five for RL (*BnaC01G0111700ZS*, *BnaC01G0111800ZS*, *BnaC01G0111900ZS*, *BnaC05G0268500ZS*, *BnaC07G0146600ZS*), eight for SFW (*BnaA05G0042600ZS*, *BnaA05G0042900ZS*, *BnaA09G0385800ZS*, *BnaA09G0386000ZS*, *BnaC02G0282600ZS*, *BnaC04G0063300ZS*, *BnaC04G0427700ZS*,*BnaC04G0521900ZS*), and five for RFW (*BnaC01G0289500ZS*, *BnaC05G0316100ZS*, *BnaC05G0316200ZS*, *BnaC08G0205400ZS*, *BnaC09G0524600ZS*).

**Table 2:** Candidate genes associated with five low nitrogen stress traits in *B. napus*.

Gene ID	AtGI	Name	Genomic position	Function	Trait	SNP
*BnaA05G0042600ZS*	*AT2G44100*	GDI1	A05:2300269.2303208	Guanosine nucleotide diphosphate dissociation inhibitor 1	SFW	scaffoldA05:2263598
*BnaA05G0042900ZS*	*AT2G44140*	ATG4A	A05:2311738.2313945	Cysteine protease ATG4a	SFW	scaffoldA05:2263598
*BnaA05G0389700ZS*	*AT3G16620*	TOC120	A05:38378520.38382168	Translocase of chloroplast 120, chloroplastic	NL	scaffoldA05:38350403/ scaffoldA05:38350423
*BnaA09G0385800ZS*	*AT3G05230*	PUB63	A09:44498527.44501543	Signal peptidase complex subunit 3A	SFW	scaffoldA09:44490242
*BnaA09G0386000ZS*	*AT3G05290*	PNC1	A09:44508204.44509694	Peroxisomal adenine nucleotide carrier 1	SFW	scaffoldA09:44490242
*BnaC01G0111700ZS*	*AT4G18190*	PUP6	C01:7553509.7556313	Probable purine permease 6	RL	scaffoldC01:7557590
*BnaC01G0111800ZS*	*AT4G18205*	PUP21	C01:7557120.7558238	Probable purine permease 21	RL	scaffoldC01:7557590
*BnaC01G0111900ZS*	*AT4G18220*	–	C01:7558757.7560053	Probable purine permease 9	RL	scaffoldC01:7557590
*BnaC01G0289500ZS*	*AT3G47960*	NPF2.10	C01:25429800.25432162	Protein NRT1/PTR FAMILY 2.10	RFW	scaffoldC01:25427138
*BnaC01G0388100ZS*	*AT1G06900*	–	C01:45002641.45003036	Insulin-degrading enzyme	SL	scaffoldC01:44994693
*BnaC02G0282600ZS*	*AT2G44100*	GDI1	C02:26881756.26882575	Guanosine nucleotide diphosphate dissociation inhibitor 1	SFW	scaffoldC02:26872986/ scaffoldC02:26959608/ scaffoldC02:26959610
*BnaC04G0063300ZS*	*AT2G45980*	ATI1	C04:5532882.5534526	ATG8-INTERACTING PRTEIN 1	SFW	scaffoldC04:5547109/ scaffoldC04:5547183
*BnaC04G0427700ZS*	*AT2G21160*	TRAP	C04:55249410.55251104	Translocon-associated protein subunit alpha	SFW	scaffoldC04:55221766/ scaffoldC04:55221778
*BnaC04G0521900ZS*	*AT5G11170*	RH15	C04:64730332.64732285	DEAD-box ATP-dependent RNA helicase 56	SFW	scaffoldC04:64681497/ scaffoldC04:64681518/ scaffoldC04:64682334/ scaffoldC04:64684991
*BnaC05G0268500ZS*	*AT1G30840*	PUP4	C05:22237214.22238386	Probable purine permease 4	RL	scaffoldC05:22262105/ scaffoldC05:22262106
*BnaC05G0316100ZS*	*AT1G47670*	AATL1	C05:30111049.30112113	Lysine histidine transporter-like 8	RFW	scaffoldC05:30136936/ scaffoldC05:30136982
*BnaC05G0316200ZS*	*AT1G47670*	AATL1	C05:30112269.30113544	Lysine histidine transporter-like 8	RFW	scaffoldC05:30136936/ scaffoldC05:30136982
*BnaC07G0146600ZS*	*AT1G28220*	PUP3	C07:27318894.27320022	Purine permease 3	RL	scaffoldC07:27322480/ scaffoldC07:27322523
*BnaC08G0205400ZS*	*AT1G14800*	–	C08:31126030.31126197	Replication protein A 70 kDa DNA-binding subunit B	RFW	scaffoldC08:31134795
*BnaC09G0524600ZS*	*AT5G13670*	UMAMIT15	C09:62391223.62392798	WAT1-related protein At5g13670	RFW	scaffoldC09:62352310/ scaffoldC09:62356206

Among these genes, several encode proteins directly involved in N uptake and assimilation. For instance, *BnaC01G0289500ZS*, encoding Protein NRT1/PTR FAMILY 2.10 (*NPF2.10*), and *BnaA05G0042600ZS*, encoding Guanosine nucleotide diphosphate dissociation inhibitor 1 (*GDI1*), are likely to participate in nitrate and peptide transport systems. These systems facilitate N redistribution and optimize N use efficiency under stress conditions.

Another prominent group includes genes encoding proteases, helicases, and transcription regulators. For example, *BnaA05G0042900ZS* encodes Cysteine protease ATG4a (*ATG4A*), which plays a role in autophagy, a crucial process for nutrient recycling under N-limited conditions. Similarly, *BnaC04G0427700ZS* encodes DEAD-box ATP-dependent RNA helicase 56 (*RH15*), potentially involved in stress-responsive RNA metabolism, while *BnaC08G0205400ZS* encodes Replication protein A 70 kDa DNA-binding subunit B, which might regulate DNA repair and replication processes under stress.

Interestingly, several genes, such as *BnaC05G0316100ZS* and *BnaC05G0316200ZS*, encode Lysine histidine transporter-like proteins (*AATL1*), suggesting an enhanced role for amino acid transporters in N remobilization during stress. Additionally, *BnaC09G0524600ZS* encodes a WAT1-related protein (*At5g13670*), which is known to regulate cell wall modification and secondary metabolism, processes that are critical for maintaining root architecture and function under LN conditions.

**Figure 5:**
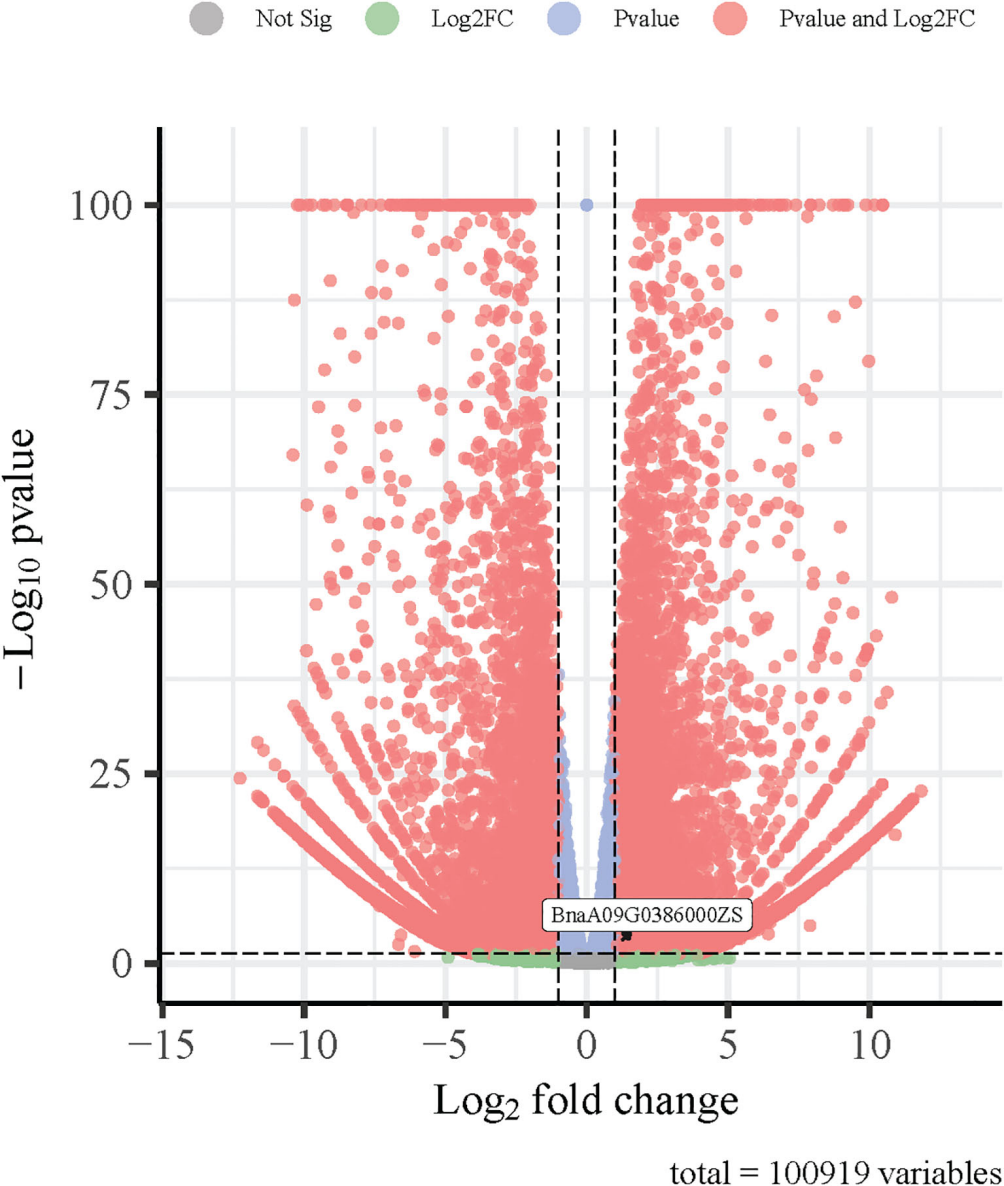
Volcano plot of gene expression changes in *Brassica napus* leaves under low-nitrogen stress.

To provide additional evidence for the involvement of the candidate genes in nitrogen stress response, we examined their expression patterns using a transcriptome dataset generated from a previous study involving low-nitrogen treatment in *B. napus*. Among the 20 candidate genes identified based on GO enrichment and trait association analysis, *BnaA09G0386000ZS* was found to be significantly upregulated under low-nitrogen conditions in both L168 and L435 lines (**Figure 5**). This upregulation suggests a potential role for *BnaA09G0386000ZS* in LN and supports its candidacy for further functional validation.

Due to differences in treatment duration and experimental design between the transcriptome study and the present research, only the expression data of the 20 candidate genes are provided in the **Supplementary materials** for reference (**Supplementary Table S7**), while the full transcriptome dataset is not included.

## 4 Discussion

Crop tolerance to LN stress is a quantitative trait regulated by multiple genes, with a clear interaction between the related genes and the external environment (Liu et al., 2021). This results in the instability of LN stress tolerance traits, which can be influenced by various factors. Therefore, precise phenotyping methods are key to obtaining accurate phenotypic data. The semi-automated hydroponic system used in this study can accommodate more than 3,000 rapeseed seedlings, including nutrient solution replenishment, nutrient solution circulation, and ventilation systems. The system maintains plants under relatively constant and uniform light, temperature, and nutrient conditions, ensuring that the obtained phenotypic data is relatively accurate and has already been applied in research (Zeng et al., 2021). Additionally, three parallel trials were conducted across three water tanks, and the multiple comparison analysis indicated no significant differences between the three trials (**Supplementary Table S5**), suggesting that the phenotypic data obtained using this system is reliable.

Under LN stress, the results of this study revealed extensive genetic variation in five phenotypic traits (NL, SL, RL, SFW, and RFW), all of which are quantitative traits controlled by multiple genes. Correlation analysis indicated that, under LN conditions, NL showed a significant positive correlation with SFW, and SFW was also significantly positively correlated with RFW. These findings suggest that RFW and SFW may be key phenotypic characteristics associated with LN tolerance. These results are consistent with previous studies, highlighting the crucial role of root growth in LN tolerance (Freschet et al., 2021; Lv et al., 2021).

The results of GWAS are influenced by factors such as species, population size, target traits, marker density, and genetic diversity within the population (Zhu et al., 2008), with population size and genetic diversity playing major roles. It is also important to note that population structure and relatedness can significantly contribute to false positives in GWAS, thus greatly affecting the accuracy of the experiment (Kichaev et al., 2019). In this study, the linear mixed model (LMM) was employed, which is particularly effective in accounting for population structure and kinship, ensuring the robustness of the results and providing a reliable foundation for further analysis. In total, 71 significant SNP markers associated with LN tolerance were detected, with several SNPs located near genes related to plant N metabolism. Based on the results of GWAS, 20 candidate genes were identified within a 50kb upstream and downstream region of the significant SNPs, all of which are implicated in N compound transport. GO enrichment analysis indicated that these genes are primarily associated with the GO term GO:0071705 (N compound transport), suggesting their potential role in N metabolism. N metabolism is crucial for plant growth and development, particularly under LN stress, where the regulation of N absorption, transport, and assimilation is essential. These identified genes are involved in the absorption of N (e.g., *NPF* family genes), its transport (e.g., *PUP* family genes), and possibly its reutilization (e.g., certain ATPases and transporters). The expression of these genes may contribute to improving N use efficiency in canola under LN conditions, thereby enhancing its tolerance to N deficiency. It is important to note that the *NRT1/PTR* (*NPF*) family comprises transporters with diverse substrate specificities, including nitrate, peptides, and phytohormones (Corratgé-Faillie and Lacombe, 2017). For instance, *BnaC01G0289500ZS*, encoding Protein NRT1/PTR FAMILY 2.10 (NPF2.10), is part of the NPF family, which includes members known to participate in nitrate uptake and transport in plants (O’Brien et al., 2016) Another prominent group includes genes encoding proteins involved in autophagy, RNA metabolism, and DNA replication. For example, *BnaA05G0042900ZS* encodes Cysteine protease ATG4a (*ATG4A*), which plays a role in autophagy, a crucial process for nutrient recycling under N-limited conditions, as reported in members of the same family in barley (Chen, 2019) and rice (Fan et al., 2020). Similarly, *BnaC04G0427700ZS* encodes DEAD-box ATP-dependent RNA helicase 56 (*RH15*), potentially involved in stress-responsive RNA metabolism (Nawaz and Kang, 2017), while *BnaC08G0205400ZS* encodes Replication protein A 70 kDa DNA-binding subunit B, which might regulate DNA repair and replication processes under stress (Capel et al., 2020). Additionally, BnaC05G0316100ZS and BnaC05G0316200ZS encode Lysine histidine transporter-like proteins (*AATL1*), suggesting an enhanced role for amino acid transporters in N remobilization during stress (Parmar et al., 2019). *BnaC09G0524600ZS* encodes a WAT1-related protein (*At5g13670*), known to regulate cell wall modification and secondary metabolism, processes critical for maintaining root architecture and function under LN conditions (Chen et al., 2021). Further research on these genes will provide valuable insights into their mechanisms of action and offer a theoretical foundation for improving N use efficiency in *B. napus*.

There have been studies utilizing GWAS methods to detect candidate genes associated with traits under LN stress in rapeseed. In this study, no significant SNP was found to be physically close to a previously reported locus Zeng et al. (2021) used 304 rapeseed inbred lines to assess plant height (PH), shoot fresh mass (SFM), leaf length (LL), and leaf width (LW) under LN stress (N: 0.3 mM). Ahmad et al. (2022) measured five root morphology traits and eight biomass-related traits in 327 *B. napus* lines under LN stress (0.5 mM). Haelterman et al. (2024) screened 300 *B. napus* inbred lines under low (N: 0.1 mM) and high (N: 5 mM) nitrate conditions, identifying 319 significant SNPs associated with 15 biomass and root morphology traits. These SNPs were subsequently integrated into 80 candidate QTLs. To further assess the genetic contribution of the candidate genes, haplotype analysis was conducted using SNPs. For some genes, no polymorphic sites were detected, and thus haplotypes could not be defined. For the remaining genes, although haplotypes were identified, most showed one haplotype or other haplotypes included only a limited number of accessions (n ≤ 6), making statistical comparisons unreliable. The haplotype classifications for all candidate genes are provided in **Supplementary Table S6**. Compared with previous studies, our GWAS identified a different set of SNPs associated with seedling-stage traits under LN stress. Unlike Ahmad et al (Ahmad et al., 2022), who integrated RNA-Seq to identify gene expression changes, our study focused on trait-based GWAS combined with haplotype analysis, revealing new candidate genes such as *NPF2.10*, *ATG4a*, and *AATL1*. Zeng et al. (2021) primarily investigated shoot traits and detected SNPs not overlapping with ours, possibly due to differences in germplasm, N levels, or sampling time points. Moreover, while Haelterman et al. (2024) concentrated on nitrate-specific root traits, our hydroponic system allowed for standardized seedling phenotyping, leading to the detection of 71 significant SNPs and 20 N-related candidate genes. To support the functional relevance of these genes, we examined their expression profiles using a previously obtained transcriptome dataset from *B. napus* seedlings subjected to LN treatment. Notably, *BnaA09G0386000ZS* was significantly upregulated under LN conditions, suggesting its involvement in nitrogen-responsive regulatory pathways. This result provides transcriptomic evidence that complements our association analysis and supports the potential role of *BnaA09G0386000ZS* in contributing to nitrogen use efficiency (NUE). However, it is important to note that the transcriptome dataset was derived from an independent experiment with a different nitrogen treatment regime and developmental stage, and thus the data were used only for reference. Further expression validation, such as qRT-PCR under comparable treatment conditions, and functional studies will be necessary to confirm the roles of this and other candidate genes in LN stress tolerance. These findings expand the current catalog of genetic variants contributing to N use efficiency in *B. napus* and offer new molecular targets for breeding.

## Data Availability

The original contributions presented in the study are included in the article/**Supplementary Material**. Further inquiries can be directed to the corresponding authors.
